# Effect of scaling on levels of interleukin 1-beta and clinical periodontal parameters among e-cigarette users and non-smokers: A prospective study

**DOI:** 10.18332/tid/189552

**Published:** 2024-07-10

**Authors:** Alaa B. Alkhalifah, Nehad T. Alfuraih, Bobby Joseph, Jagan K. Baskaradoss

**Affiliations:** 1 College of Dentistry, Kuwait University, Kuwait City, State of Kuwait; 2 Oral Health Centre of Western Australia, The University of Western Australia Dental School, Nedlands, Australia; 3 Department of Developmental and Preventive Sciences, College of Dentistry, Kuwait University, Kuwait City, State of Kuwait

**Keywords:** cigarette, electronic, gingivitis, interleukins, vaping

## Abstract

**INTRODUCTION:**

This cohort study aimed to compare the effect of ultrasonic scaling on the expression of IL-1β in the gingival crevicular fluid (GCF) among ENDS users and non-smokers (NS) with gingivitis.

**METHODS:**

Self-reported current electronic nicotine delivery system (ENDS) users and NS with generalized gingivitis were included in this study. All the patients underwent scaling at the baseline visit (T0). Clinical measures, periodontal parameters [probing depth (PD), plaque index (PI), and bleeding on probing (BOP)], and GCF IL-1β were measured at T0, after 1 week (T1) and after 3 weeks (T2). Wilcoxon signed rank test was used to assess the changes in the periodontal measurements and IL-1β levels at different time points and Mann–Whitney U Test was used to compare the two groups.

**RESULTS:**

A total of 38 individuals (18 NS and 20 ENDS users) participated in the study. The PD was significantly higher in ENDS users than in NS at baseline. However, the PI and BOP were similar in all groups at baseline. At T1, the PI was significantly lower for NS than for ENDS users (p=0.045). At T2, there were no significant differences in any of the parameters assessed between the two groups. For ENDS users, BOP was significantly lower at T1 than at baseline. For NS, the BOP at T1 and T2 and the PI at T1 were significantly lower than at baseline. There was no difference in the GCF IL-1β levels in NS and ENDS users at baseline, T1, and T2. At T2, there was a significant reduction in IL-1β (p<0.05) than at baseline in both groups.

**CONCLUSIONS:**

Both ENDS users and NS with gingivitis responded similarly to scaling. GCF IL-1β levels were significantly higher at baseline (p<0.05) compared with their levels at T1 and T2 for both the groups.

**CLINICAL TRIAL REGISTRATION:**

The study was registered on the official website of ClinicalTrials.gov

**IDENTIFIER:**

ID NCT05745324

## INTRODUCTION

The World Health Organization estimates that tobacco use currently causes approximately six million deaths worldwide annually^1^. According to the American Lung Association, smoking is the cause of 90% of all lung cancers. Waterpipes, cigars, and smokeless tobacco are other known forms of tobacco smoking. The detrimental effects of tobacco smoking on general and oral health have been well documented in the literature^2,3^. In terms of general health, the effects of tobacco smoking are systemic and more pronounced in the cardiovascular, respiratory, and immune systems^3^.

Although cigarette smoking (CS) is the most common form of smoking, electronic nicotine delivery systems (ENDS) have recently been gaining popularity, particularly among younger age groups. A nationally representative survey in the United States reported that ENDS have been the most frequently used tobacco products since 2014^4^ among youths aged 12–17 years.

Although the effects of CS on the periodontium have been thoroughly studied, limited studies have compared the effects of ENDS on the periodontium^5^. Similar to tobacco smoking, the use of ENDS is detrimental to various bodily systems, although the evidence supporting this is limited^6^. As the oral cavity is the first site to be exposed to tobacco smoke and ENDS aerosols, the deleterious effects on oral health are widely prominent^5^. ENDS have comparable or even higher concentrations of nicotine than CS and therefore may exert a similar vasoconstrictive activity on gingival blood vessels concomitant with resultant damage to gingival fibroblasts^7^. In contrast, St Helen et al.^8^ reported that systemic nicotine exposure was, on average, lower with single use of e-cigarettes as compared with conventional cigarettes smokers.

Gingivitis is a site-specific inflammatory condition initiated by dental biofilm (plaque) accumulation and characterized by gingival redness and edema and the absence of periodontal attachment loss^9^. Plaque-induced gingivitis is a reversible condition, where the tissue alterations are reversed once the dental plaque is removed. However, presence of gingivitis is clinically significance because it is considered the precursor of periodontitis, which involves progressive connective tissue attachment and bone loss. Although not every case of gingivitis progresses to periodontitis, managing gingivitis is considered the first step towards the prevention of periodontitis^9^.

Of the most important humoral factors influencing immuno-inflammatory reactions on the periodontal tissues is IL-1β, a commonly analyzed biomarker when studying the inflammatory response of the periodontium to smoking^10^. IL-1β promotes development of an inflammatory response, amplifies inflammation, and modulates various immunological processes. It stimulates fibroblast proliferation, prostaglandin E2 production, and activates the release of matric metalloproteinases from different cell populations, leading to the degradation of extracellular matrix proteins. This cytokine also promotes osteoclast formation and is a potent inducer of bone demineralization^11^.

A recent study reported no difference in whole salivary IL-1β levels between CS and ENDS users after 12-weeks of non-surgical periodontal therapy (NSPT)^12^. AlMubarak et al.^13^ compared salivary IL-1β levels among young adults involuntarily exposed to vapors from ENDS with those of unexposed individuals. Significantly higher levels of IL-1β were observed among the exposed compared with unexposed participants. IL-1β exhibits high sensitivity and specificity for discriminating between subjects with gingivitis and healthy subjects^14^. IL-1β is reportedly one of the best predictive biomarkers for periodontal diseases^15,16^.

Regionally, a recent study observed that the rates of current cigarette smokers among Kuwait University students were the highest in comparison to other Arabian Gulf countries^17,18^. Another study found that almost half of the male students at Kuwait University were cigarette smokers^19^. Moreover, half of the Kuwaiti men have smoked tobacco at some point in their life^20^. Interestingly, a recent study conducted in Kuwait reported that ENDS, rather than CS, is more prevalent among adolescents^21^. To date, no published studies have investigated the effects of ENDS in young adults with generalized gingivitis. There is a paucity of evidence comparing the effect of ultrasonic scaling on the expression of inflammatory biomarkers, like IL-1β, between ENDS users and non-smokers (NS) among young adults with gingivitis. The present study is based on the null hypothesis that the expression of IL-1β after ultrasonic scaling would be similar between ENDS users and NS. The aim of the present study was to study the soft tissue and inflammatory response to ultrasonic scaling among ENDS users and NS by comparing the levels of IL-1β in the gingival crevicular fluid (GCF).

## METHODS

### Study area and setting

This prospective cohort study was conducted between November 2021 and September 2022 at Kuwait University Dental Center (KUDC). A convenience sample was selected from regular patients who attended KUDC. The investigators involved in clinical and laboratory investigations and statistical analyses were blinded to the vaping status of the participants.

### Selection criteria

The inclusion criteria were as follows: 1) ENDS users or non-smokers, 2) aged 18–25 years, 3) generalized gingivitis, and 4) having a minimum number of 20 teeth. Patients who underwent professional dental cleaning within the past 3 months; CS; dual smokers (CS individuals and ENDS users or other forms of tobacco); and patients with cardiovascular, hepatic, endocrine, and/or renal diseases, were excluded.

### Ethics

Ethical approval was obtained from the Ethics Committee of the Health Sciences Center of Kuwait University (approval: 04.10.2021; protocol number: VDR/ED/14). This study was conducted according to the principles outlined in the Declaration of Helsinki on Human Medical Experimentation. Participants were required to read and sign a consent form. Before signing the consent form, all participating patients were informed that they could withdraw from the study at any stage without penalty and were invited to ask questions. The manuscript is presented in accordance with the Strengthening the Reporting of Observational Studies in Epidemiology (STROBE)^22^.

### Study participants and grouping

**Figure 1:**
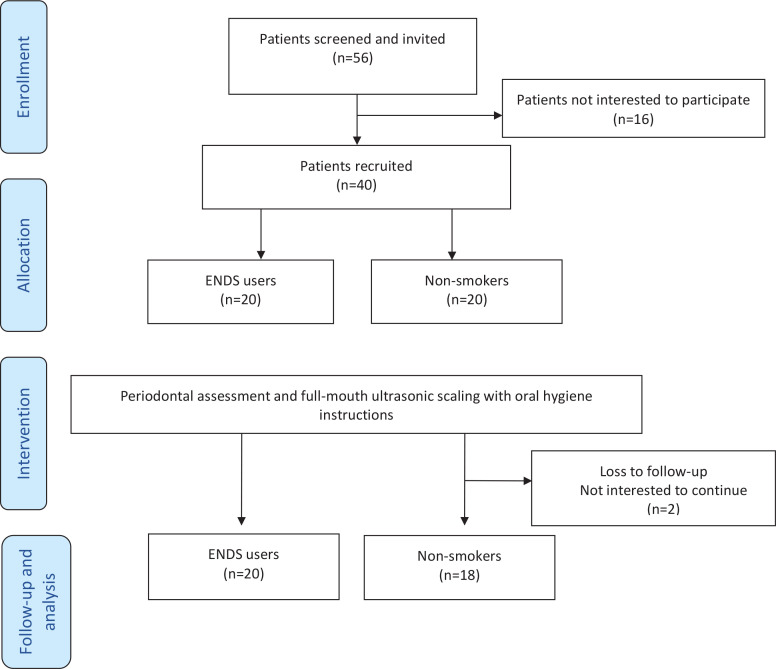
Flow diagram of study participants

This study recruited 20 current ENDS users and 20 NS (Figure 1). All participants were requested to refrain from eating and using any oral hygiene methods for at least 3 h prior to their visit. Non-smokers were defined as those who had never smoked cigarettes in their lives or used any nicotine-containing product within the preceding 5 days^23^. ENDS users were defined as those who had never used conventional cigarettes and had been using any electronic nicotine products for at least one session per day for the past 3 months. Electronic products included e-cigarettes, e-cigars, e-pipes, e-hookahs, and personal vaporizers, as well as battery-powered vape pens and hookah pens. A patient was categorized as having ‘generalized gingivitis’ if the patient presented with a bleeding on probing (BOP) score of >30% without attachment loss or radiographic bone loss^9^.

### Questionnaire

A standardized questionnaire was used to gather information regarding sex, age, nationality, marital status, education level, oral hygiene status, last dental visit, and the most recent professional dental cleaning, smoking status, type of smoked product, duration of ENDS use (in years), frequency of ENDS use per day, number of ENDS puffs taken per session, family smoking history, exposure to secondhand smoke, general health status, and attitude towards smoking.

### Clinical procedure and sample collection

At baseline (T0), all participants underwent full-mouth ultrasonic scaling, which took approximately 60–90 min. Scaling consisted of plaque and calculus removal using an ultrasonic scaler (Satelec P-5 Booster) set at different power modes with or without the use of sterile Gracey curettes (Hu-Frieddy, Chicago, IL, USA). Subsequently, routine oral hygiene maintenance protocols were implemented. Patients were recalled 1 week (T1) and 3 weeks (T2) after baseline.

### Clinical examination

For all participants, the Löe & Silness gingival index (GI) and Silness & Löe plaque index (PI), BOP, and periodontal pocket depth (PD) were measured on the mesiobuccal, midbuccal, distobuccal, distolingual/palatal, mid-lingual/palatal, and mesio-lingual/palatal surfaces of all maxillary and mandibular teeth by two trained and calibrated examiners (intra- and inter-rater reliability Kappa scores were >70% for all clinical measures). PD measurements were recorded to the nearest mm using UNC-15 periodontal probe (Hu-Friedy, Chicago, IL., USA) using a light force (approximately 0.3 N).

### GCF sample collection

At baseline (T0), GCF samples were obtained prior to scaling from the deepest pocket on the buccal side of the first or second molars, as described in an earlier study^24^. Briefly, the selected site was isolated with sterile cotton rolls, and the supragingival oral biofilm was gently removed prior to sample collection. A three-way syringe was used to dry the tooth, and a sterile paper strip (Periopaper, Amityville, NY, USA) was inserted into the pocket for 30 s. A calibrated digital machine (Periotron 8000, Oraflow Inc., Plainview, NY, USA) was used to measure the volume of GCF. Samples contaminated with blood were discarded, and another site was used for GCF sample collection. The strips were placed in 300 μL 0.01 M phosphate-buffered saline (pH 7.2) in Eppendorf tubes (ep TIPS Standard, Eppendorf AG, Hamburg, Germany). GCF samples were obtained in the same manner after 1 week (T1) and 3 weeks (T2). The samples were immersed in 0.5 mL sterile distilled water in Eppendorf tubes (ep TIPS Standard, Eppendorf AG, Hamburg, Germany). GCF samples were obtained in the same manner after 1 week (T1) and 3 weeks (T2). IL-1β levels in GCF were analyzed using human interleukin-1-beta/interleukin-1p-F2 Quantikine ELISA kits from R&D Systems (Minneapolis, MN, USA)^25^.

### Power and statistical analysis

The sample size was determined using the computer software G*Power (version 3.0.10; Franz Faul Universitat, Kiel, Germany) based on values from a previous study^24^. For an assumed estimated effect size of 2.5, it was determined that a sample size of 18 individuals/group would be required to achieve 95% power to detect the difference in IL-1β levels between ENDS users and NS.

The Kolmogorov–Smirnov test was used to assess data normality. As the data were skewed, independent sample Mann–Whitney U Test was used to compare the periodontal status and IL-1β levels between ENDS users and NS. Wilcoxon signed rank test was used to assess the changes in the periodontal measurements and IL-1β levels at different time points. Bonferroni adjustments were applied for multiple comparisons. The level of significance was set at p<0.05. All analyses were performed using SPSS 27.0 (IBM Corp. Released 2017. IBM SPSS Statistics for Windows, Version 27.0. Armonk, NY: IBM Corp.).

## RESULTS

### Demographics

Forty subjects (NS=20 and END users=20) consented to participate in this study, of which two NS were lost to follow-up after the first visit. The demographic data of the 38 subjects are presented in Table 1. The mean age of END users was 22.4 years and that of NS was 22.6 years. The majority of the participants (n=32) were Kuwaitis, held Bachelor’s degrees (n=28), and were unmarried (n=30). More than half of the respondents brushed only once daily (n=25) and did not use any other dental aids.

**Table 1:** Demographic characteristics of the study cohort

*Characteristics*	*ENDS users (N=20) n (%)*	*Non-smokers (N=18) n (%)*
**Sex** (Male)	20 (100)	18 (100)
**Mean Age** (years)	22.4	22.6
**Nationality**		
Kuwaiti	14 (70.0)	18 (100)
Non-Kuwaiti	6 (30.0)	0 (0.0)
**Marital status**		
Single	15 (75.0)	15 (83.3)
Married	5 (25.0)	3 (16.7)
**Education level**		
Bachelor’s	17 (85.0)	11 (61.1)
High school or lower	3 (15.0)	7 (38.9)
**Brushing frequency**		
Once daily	13 (65.0)	12 (66.7)
≥2 times daily	5 (25.0)	3 (16.7)
Irregularly	2 (10.0)	3 (16.7)
**Use of additional dental aids**		
Mouthwash only	3 (15.0)	6 (33.3)
Mouthwash and floss	5 (25.0)	3 (16.7)
Floss only	2 (10.0)	4 (22.2)
None	10 (50.0)	5 (27.8)
**Last dental visit** (months prior)		
3–6	8 (40.0)	9 (50.0)
6–12	6 (30.0)	4 (22.2)
>12	6 (30.0)	5 (27.8)
**Last scaling done** (months prior)		
3–6	4 (20.0)	8 (50.0)
6–12	11 (55.0)	5 (27.8)
>12	5 (25.0)	5 (27.8)

### Smoking history

All ENDS users in this study had been vaping for at least 1 year. Approximately half of the ENDS users vaped for 1–3 sessions per day. Most participants agreed that smoking negatively impacted both general and oral health and was a major risk factor for oral cancer (Table 2).

**Table 2:** Smoking history of participants

	*ENDS users (N=20) n (%)*	*Non-smokers (N=18) n (%)*
**Years of vaping**		
1–3	19 (95.0)	
>3	1 (5.0)	
**Sessions of vaping per day**		
>6	6 (30.0)	
4–6	5 (25.0)	
1–3	9 (45.0)	
**Number of puffs per session**		
>100	3 (15.0)	
20–100	7 (35.0)	
10–20	7 (35.0)	
<10	3 (15.0)	
**Smoking family members**		
None	11 (55.0)	5 (27.8)
1	1 (5.0)	4 (22.2)
2	5 (25.0)	5 (27.8)
≥3	3 (15.0)	4 (22.2)
**Where they smoke**		
Outside the house	7 (35.0)	8 (44.4)
Inside the house	2 (10.0)	2 (11.1)
Outside and inside the house	0	3 (16.7)
**Exposure to secondhand smoking**		
No exposure	15 (75.0)	7 (38.9)
Workplace and home	1 (5.0)	3 (16.7)
Workplace only	2 (10.0)	7 (38.9)
Home only	2 (10.0)	1 (5.6)
**Smoking affects your general health**		
Strongly agree/agree	19 (95.0)	18 (100.0)
Neutral/strongly disagree/disagree	1 (5.0)	0 (0.0)
**Smoking affects your oral health**		
Strongly agree/agree	18 (90.0)	17 (94.4)
Neutral/strongly disagree/disagree	2 (10.0)	1 (5.6)
**Smoking is a risk factor for oral cancer**		
Strongly agree/agree	16 (80.0)	16 (88.9)
Neutral/strongly disagree/disagree	4 (20.0)	2 (11.1)
**Smoking complicates dental treatment**		
Strongly agree/agree	15 (75.0)	15 (83.3)
Neutral/strongly disagree/disagree	5 (25.0)	3 (16.7)
**Smoking has a role in dental treatment failure**		
Strongly agree/agree	13 (65.0)	11 (61.1)
Neutral/strongly disagree/disagree	7 (35.0)	7 (38.9)
**Smoking compromises health after tooth extraction**		
Strongly agree/agree	14 (70.0)	12 (66.7)
Neutral/strongly disagree/disagree	6 (30.0)	6 (33.3)
**Smoking causes teeth discoloration**		
Strongly agree/agree	18 (90.0)	16 (88.9)
Neutral/strongly disagree/disagree	2 (10.0)	2 (11.1)
**Smoking causes bad breath**		
Strongly agree/agree	18 (90.0)	18 (100.0)
Neutral/strongly disagree/disagree	2 (10.0)	0 (0.0)

### Clinical periodontal parameters

At baseline, the PD was significantly higher in ENDS users than in NS (p=0.021). BOP and PI were similar between the groups at baseline. At T1, the PI was significantly lower for NS users than for ENDS users (p=0.045). At T2, there were no significant differences in any of the parameters assessed between the two groups. For ENDS users, bleeding on probing was significantly lower at T1 than at baseline. For non-smokers, the bleeding on probing at T1 and T2 and the plaque index at T1 were significantly lower compared to baseline (Table 3).

**Table 3:** Periodontal status at baseline and follow-up visits

*Parameters*	*T0 (Baseline)*			*T1 (After 1 week)*			*T2 (After 3 weeks)*		
	*ENDS users (N=20)*	*Non-smokers (N=18)*	p*	*ENDS users (N=20)*	*Non-smokers (N=18)*	p*	*ENDS users (N=20)*	*Non-smokers (N=18)*	p*
Periodontal probing depth (mm)	1.91 ± 0.3	1.75 ± 0.2	0.021	1.64 ± 0.5	1.56 ± 0.5	0.306	1.42 ± 0.8	1.61 ± 0.4	0.173
Bleeding on probing (%)	20.2 ± 15.7	15.1 ± 11.8	0.132	11.3 ± 10.5^†^	7.30 ± 7.9^‡^	0.097	8.28 ± 8.1	7.03 ± 6.0^‡^	0.298
Plaque index	0.54 ± 0.2	0.43 ± 0.3	0.085	0.32 ± 0.2	0.21 ± 0.1^‡^	0.045	0.22 ± 0.2	0.23 ± 0.1	0.456

* Independent sample Mann–Whitney U Test comparing ENDS users with Non-smokers.

† Statistically significant (p<0.05) compared to baseline values of ENDS users using Wilcoxon Signed Rank Test.

‡ Statistically significant (p<0.05) compared to baseline values of Non-smokers using Wilcoxon Signed Rank Test.

### GCF IL‑1b levels

The mean GCF volume (µL) for ENDS users and NS were 0.8 ± 0.3 and 0.6 ± 0.1, respectively. The mean IL-1β values were 131.99 ± 88.2, 59.72 ± 50.5, and 67.73 ± 39.9 at baseline, T1 and T2, respectively (Table 4). IL-1β levels were significantly higher in both groups at baseline (p<0.05) than at T1 or T2. There was no difference in GCF IL-1β levels among ENDS users and NS at baseline, T1, and T2. There was no difference in GCF IL-1β levels between patients with different demographic characteristics (data not shown).

**Table 4:** Interleukin 1-beta levels at baseline and follow-up visits of ENDS users and NS

*Parameter*	*T0 (Baseline)*				*T1 (After 1 week)*				*T2 (After 3 weeks)*			
	*Total*	*ENDS users (N=20)*	*Non-smokers (N=18)*	*p ^§^*	*Total*	*ENDS users (N=20)*	*Non-smokers (N=18)*	*p ^§^*	*Total*	*ENDS users (N=20)*	*Non-smokers (N=18)*	*p ^§^*
IL-1β (pg/mL)	131.99 ± 88.2	147.32 ± 98.1	114.96 ± 74.8	0.27	59.72 ± 50.5*	55.83 ± 53.8*	64.06 ± 47.64*	0.62	67.73 ± 39.9*	67.81 ± 44.9*	67.64 ± 34.7*	0.99

* Significant difference (p<0.05) compared to baseline values within the groups (Related Samples Wilcoxon Signed Rank Test).

§ Mann–Whitney U Test.

## DISCUSSION

To the best of our knowledge, this is the first study comparing the effect of ultrasonic scaling on the expression of IL-1β levels and clinical periodontal parameters between ENDS users and NS in a young adult population.

The effect of smoking on the periodontium occurs owing to changes in the microflora, host response to bacterial challenges, or a combination of both^26^. Studies have demonstrated that tobacco smoking creates an environment conducive to the colonization of subgingival periodontal pathogens, forming at-risk sites for future periodontal conditions by altering the host-bacterial interaction^27^. Studies have shown that tobacco smoking induces immune dysregulation^28^. Neutrophils exhibit alterations in chemotaxis, phagocytosis, and oxidative burst^29^. In addition, studies have concluded that immunoglobulin G2 is reduced in smokers compared to NS with periodontitis, suggesting that smokers are more susceptible to periodontal bacteria^30^. Although these mechanisms have been mentioned in the literature, the detrimental effects of smoking on the periodontium are multifactorial and not clearly understood; therefore, further studies are needed.

IL-1β levels in the GCF were similar between groups at different time points. This finding is similar to that of a recent study, which found no difference in clinical periodontal parameters after NSPT^12^. BinShabai et al.^24^ also reported no statistically significant difference in clinical periodontal parameters and GCF proinflammatory cytokine levels between ENDS users and NS. The authors suggested a relatively short duration of vapor as a possible explanation^24^. Nevertheless, the same study reported an increased level of periodontal inflammatory parameters and GCF cytokines among ENDS users compared with that of NS users. Our study demonstrated that the levels of IL-1β were significantly higher in both groups at baseline (p<0.05) than at T1 and T2, and the difference was not significant between ENDS users and NS at each time interval. Our study found that at T1, the plaque index was significantly lower for NS than for ENDS users. Some studies have found that smoking was positively correlated with IL-1β levels^31^. Conversely, a study has found that smoking was negatively correlated with IL-1β levels, although the results were not statistically significant^32^. No relationship between smoking and IL-1β levels have also been reported in the literature^33^. There were no female participants in the study. Studies have shown that hormonal fluctuations during menstruation are associated with an increased expression of destructive inflammatory cytokines including IL-1 β^34^. Influence of hormonal fluctuations was minimized in this study.

Similar to results of this study, Alhumaidan et al.^12^ have reported no difference in the periodontal parameters after non-surgical periodontal therapy between ENDS users and cigarette-smokers. The same study reported no significant difference in salivary IL-1β levels at baseline and 12-weeks of follow-up between the two groups. According to recent studies, tobacco smoking causes oral vascular changes, periodontal diseases, dry sockets, Candida infections, impaired inflammatory responses, and oral cancer^35^. ENDS have been reported to cause oral mucosal lesions, periodontitis, and impaired inflammatory response^5^. Recent studies have concluded that nicotine stomatitis, hairy tongue, and angular stomatitis are more common in ENDS users than in former smokers and NS^10^. A recent systematic review of *in vitro* studies reported that nicotine, at levels found in tobacco smokers, nicotine replacement therapy users and e-cigarette users, is unlikely to be cytotoxic to human gingival and periodontal cells^36^. It may be presumed that substances other than nicotine may be exhibiting detrimental effects on the periodontal health. Additionally, it may be hypothesized that individuals with a long-term history of vaping may have poorer gingival health status and exhibit significantly higher levels of proinflammatory cytokines in the GCF compared with individuals with a shorter history of vaping.

### Limitations

The present study has several limitations. First, it did not include CS or dual smokers. This limits the ability of this study to compare the inflammatory and clinical parameters between these groups, which are relatively more common. Second, self-reported outcomes rely on the self-recall abilities of patients, which may have significantly introduced bias and affected the results. Third, only one inflammatory marker was used in our study because of lack of funds, and it may be recommended for future studies to use multiple markers from different domains of the inflammatory cascade to better understand this relationship. Furthermore, no additional diagnostic tools such as radiographs or clinical photographs were used, which could have improved the accuracy of the clinical measurements. Finally, inability to perform formal assessment of interaction terms or adjust for residual confounding that may have resulted from non-controlled adjustments may have influenced the study results. Convenience sampling method adopted in this study limits the generalizability of the study findings to other populations.

Future longitudinal studies among CS and ENDS users with and without periodontitis may help document the inflammatory responses to ultrasonic scaling. In view of the findings of this study, although gingiva responded similarly between ENDS users and NS, it is prudent to conclude that nicotine intake in any form should be strongly discouraged, especially in the younger population.

## CONCLUSIONS

This study concluded that there is no significant difference in the changes in the level of IL-1β at baseline and 3-weeks of follow-up between the two groups. In addition, the clinical periodontal parameters among ENDS users and NS were similar at the end of the study period. In both ENDS users and NS with gingivitis, GCF IL-1β levels were significantly higher at baseline (p<0.05) than at T1 and T2; however, the difference was not significant between ENDS users and NS at each time interval. In addition, at T1, the plaque index was significantly lower in the NS than in the ENDS users.

## Supplementary Material



## Data Availability

The data supporting this research are available from the authors on reasonable request.
